# Implications of dimeric activation of PDE6 for rod phototransduction

**DOI:** 10.1098/rsob.180076

**Published:** 2018-08-01

**Authors:** Trevor D. Lamb, Martin Heck, Timothy W. Kraft

**Affiliations:** 1 Eccles Institute of Neuroscience, John Curtin School of Medical Research, The Australian National University, Australian Capital Territory 2600, Australia; 2 Institut für Medizinische Physik und Biophysik der Charité, Universitätsmedizin Berlin, corporate member of Freie Universität Berlin, Humboldt-Universität zu Berlin, and Berlin Institute of Health, Berlin, Germany; 3 Department of Optometry and Vision Science, University of Alabama at Birmingham, Birmingham, AL, USA

**Keywords:** rod photoreceptors, phototransduction, phosphodiesterase PDE6, transducin, dimeric activation, response kinetics

## Abstract

We examine the implications of a recent report providing evidence that two transducins must bind to the rod phosphodiesterase to elicit significant hydrolytic activity. To predict the rod photoreceptor's electrical response, we use numerical simulation of the two-dimensional diffusional contact of interacting molecules at the surface of the disc membrane, and then we use the simulated PDE activity as the driving function for the downstream reaction cascade. The results account for a number of aspects of rod phototransduction that have previously been puzzling. For example, they explain the existence of a greater initial delay in rods than in cones. Furthermore, our analysis suggests that the ‘continuous’ noise recorded in rods in darkness is likely to arise from spontaneous activation of individual molecules of PDE at a rate of a few tens per second per rod, probably as a consequence of spontaneous activation of transducins at a rate of thousands per second per rod. Hence, the dimeric activation of PDE in rods provides immunity against spontaneous transducin activation, thereby reducing the continuous noise. Our analysis also provides a coherent quantitative explanation of the amplification underlying the single photon response. Overall, numerical analysis of the dimeric activation of PDE places rod phototransduction in a new light.

## 1. Introduction

Three key proteins mediating activation of the light response in rod photoreceptors are located in the disc membranes of the cell's outer segment, and comprise rhodopsin (a G-protein-coupled receptor), transducin (a heterotrimeric G-protein) and the PDE (a heterotetrameric cyclic GMP phosphodiesterase, PDE6). The fourth key protein, the cyclic nucleotide-gated ion channel (CNGC), is located in the plasma membrane that envelops the outer segment discs. In addition, a number of other proteins mediating recovery of the light response and light adaptation are also located in the disc or plasma membranes. The interaction between the participating proteins occurs primarily via contact resulting from their lateral diffusion in the disc membrane, or at its surface. When rhodopsin is isomerized by the absorption of a photon, it enters an active state (denoted R*) that is able to catalyse the activation of transducin, leading to the formation of G*α* · GTP (denoted G*). G*, in turn, activates the third protein, the PDE, and it is the stoichiometry of that mechanism that is the focus of this paper. Upon activation, the role of the PDE is to hydrolyse cyclic GMP (cGMP) in the cytoplasm, whereupon the lowered cytoplasmic cGMP concentration triggers the closure of the ion channels, thereby generating the cell's electrical response to light.

Quantitative descriptions of the molecular mechanisms underlying vertebrate phototransduction assume that the rod PDE (comprising PDE6*α* and PDE6*β* with two identical PDE6*γ* subunits) behaves as a pair of independent catalytic subunits [1]. However, contrary to this idea, evidence was presented as long ago as 1989 that the activation of PDE by transducin does not, in fact, occur independently [2], and recent work by Qureshi *et al*. has provided compelling evidence that the activation of the PDE indeed exhibits pronounced functional asymmetry [3,4]. This work has shown that the binding of a single molecule of active transducin (G*) produces a form (PDE*) with negligible hydrolytic activity, whereas binding of the second G*, to form PDE**, causes full activation. One major advantage of this arrangement for the rod is that it provides immunity against noise generated by random activation of the PDE triggered by spontaneous thermal activation of G* [3–5]. This protection is conferred because it is only upon the concerted activation of multiple G*s, catalysed by an isomerized rhodopsin molecule (R*), that there is a sufficiently high local concentration of G* for any given molecule of G* to be able to ‘find’ a singly bound PDE* to bind to, and thereby activate it to PDE**.

It has been shown that the PDE6*α* and PDE6*β* catalytic subunits have closely comparable hydrolytic activity when expressed as chimaeric homodimers [6], yet the same study showed a much weaker steady-state affinity of transducin for the heterodimeric rod PDE6, in comparison with those rod-like homodimers (or the cone homodimer). Indeed, the affinity of approximately 1 µM transducin for the rod PDE6 reported in that study is similar to the weaker affinity reported by Qureshi *et al*. [4] for their membrane-bound preparation.

In this paper, we investigate the implications that a model of dimeric activation of PDE** has for the predicted electrical responses of rod photoreceptors. By numerically simulating the lateral diffusion of the interacting molecules at the disc membrane, we are able to predict the kinetics of PDE** activation, and to contrast this with the conventional case that is predicted on the standard model of independent PDE subunits. We show, first, that the rate of formation of fully activated PDE** molecules is considerably lower than simply half the rate at which G* is activated by an isomerized rhodopsin (R*), and we use this information to provide a new and coherent set of parameters to describe the amplification underlying the single-photon response. Second, we find that a significant delay (of approx. 7 ms) occurs, prior to the ramp-like rise in PDE** concentration, and we show that the existence of this delay resolves an apparent paradox that was recently reported by Rotov *et al.* [7]. Third, we analyse the unitary electrical responses that are evoked by the random activation of PDE**s, and show that the continuous noise that has been reported in mouse rods is consistent with spontaneous activation of unitary PDE** events at a mean rate of approximately 10–40 PDE** events s^−1^ per rod. On the assumption that these PDE** events are triggered by transducin, we estimate the rate of spontaneous activation of transducin molecules to be far higher, at approximately 2500–4500 G* events s^−1^ per rod, with the average response to each transducin activation being far smaller than that induced by each PDE**.

## 2. Model, theory and methods

### 2.1. Model of molecular interactions between transducin and PDE6

As proposed by Qureshi *et al.* [3,4], our model invokes dimeric activation of PDE6 by transducin. Thus, binding of a first G* to form G*-PDE (denoted here as PDE*) is assumed to result in negligible hydrolytic activity, and the binding of a second G* to form G*-PDE-G* (denoted PDE**) is required for the attainment of full hydrolytic activity. In order to keep our model as simple as possible, we will not, at this stage, invoke any physical asymmetry within the PDE6 molecule (e.g. between the *α* and *β* subunits), nor will we specify whether either of these subunits is preferentially the first to bind a G*. Likewise, we will not specify the exact role of the two *γ* subunits in the interaction or in activation. These are matters that could be addressed in the subsequent development of the model, when more detailed information is available at a molecular level. In our current version, we simply specify that the singly bound PDE* molecule has minimal activity compared with the doubly bound PDE** molecule, as reported by Qureshi *et al.* [4], who found its fractional activity to be less than 2.5%.

**Figure 1.**
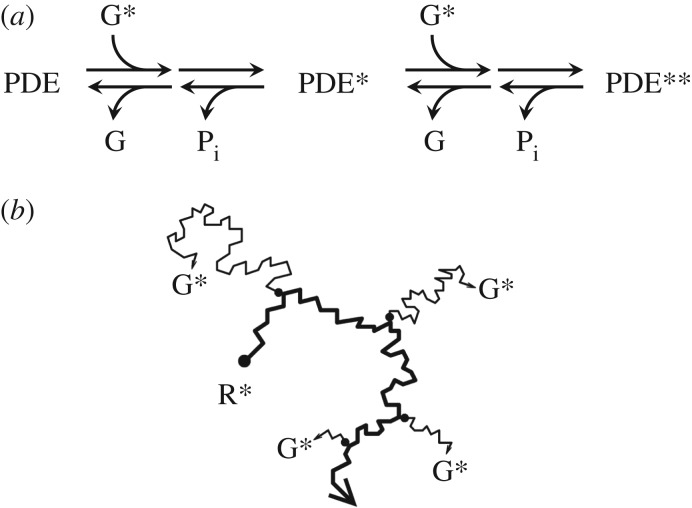
Models of dimeric activation of PDE6 and lateral diffusion of molecules. (*a*) Schematic of PDE reactions. Rightward arrows show activation steps, and leftward arrows show shut-off steps. An activated transducin (G*) can bind either to a PDE or to a PDE*, and the bound form then transitions to PDE* or PDE**, respectively; in this paper, the transition is assumed to occur instantaneously (rate = ∞). Shut-off reactions occur stochastically through GTPase activity (with release of phosphate, P_i_) followed by dissociation of the GDP-bound transducin (G). The rate constant of stochastic GTPase activity is denoted *k*_E*_ or *k*_E**_ in the two cases; the subsequent dissociation is assumed to occur instantaneously (rate = ∞). (*b*) Lateral diffusion of molecules at the disc surface. A single activated rhodopsin molecule (R*) is shown diffusing laterally in the disc membrane (bold line) from its initial position (larger filled circle). At each of the locations indicated by the smaller filled circles, a transducin molecule is activated (to G*) and diffuses laterally at the membrane surface. For simplicity, we have not attempted to illustrate contact with PDE molecules.

The postulated interactions of the PDE6 heterotetramer with transducin are illustrated schematically in figure 1*a*, where the states of the molecule with 0, 1 and 2 transducins bound are indicated by PDE, PDE* and PDE**, respectively. The rightward arrows denote activation steps, and the leftward arrows denote shut-off steps. Furthermore, the possibility of various transition states (such as ‘bound but not yet activated’) is envisaged by the head-to-tail arrows, but again will not be considered here. Contact between PDE and a first G* opens the possibility of their binding, at some rate constant; the diffusion limit to this step occurs when that rate constant approaches infinity. Thereupon (and possibly following some transition state), the molecule is in the PDE* configuration, and for our initial analysis, we assume that this state has no hydrolytic activity. Contact between PDE* and a second G* likewise opens the possibility of their binding, at some rate constant. With two G*s bound (and again following the possibility of some transition state), the PDE** configuration has its full hydrolytic activity, at a level that has been determined in biochemical experiments. Each of the G*-bound states (PDE* and PDE**) decays via hydrolysis of the G*'s terminal phosphate (i.e. by GTPase activity, accelerated by RGS9). Here we assume that the decay of PDE** returns it to the PDE* state, and furthermore that the decay of PDE* returns it to the resting PDE state.

To estimate the kinetics of formation of PDE* and PDE**, we simulated the lateral diffusion of molecules in the plane of the disc membrane, as sketched schematically in figure 1*b*, using the approach described previously [8], with a custom computer program described in the next section. Intermolecular reactions are assumed to occur at the diffusion limit (i.e. upon every diffusional contact). Inactivation of the active molecular species is assumed to occur as follows. For R*, shut-off occurs stochastically according to the kinetic scheme reported recently [9]. For activated transducin, shut-off occurs rapidly when the G* is bound to PDE and in the presence of RGS9, but at a lower rate when it is unbound. In our simulations, we assume that shut-off occurs only when the G* molecule is bound as either the PDE* state or the PDE** state, and that it then occurs stochastically at a defined mean rate. Thus, we begin by ignoring the decay of free G*; this is justified at intensities causing no more than one R* per disc surface, because the level of free G* is small (see Results). We assume that, as PDE** has two G*s bound, it decays at twice the rate that PDE* decays. We also assume that this GTPase reaction causes the immediate transition of PDE** to PDE*, or of PDE* to PDE, as the case may be; this is indicated by the leftward arrows in figure 1*a*. The standard values that we adopted for all the parameters of the reactions at the disc membrane are listed in table 1.

**Table 1.** Standard parameters for simulation of lateral diffusion reactions underlying dimeric activation of PDE**. The stimulus corresponded to a brief flash that delivered a single photoisomerization. Thus, each simulation began at *t* = 0, with a single R* activated at a random location on the circular disc, with zero delay. The number of PDE holomers was the same in each trial (i.e. it was not a stochastic variable). The density of transducin molecules is not required, because the ‘shortcut’ method generated G*s stochastically at the specified rate, *ν*_RG_ (see text). Reactions between diffusing molecules occurred at the simulated diffusion limit (i.e. upon each contact between molecules that could react with each other).

symbol	description	value	units
	*dimensions and time increment*		
*d*	diameter of circular disc	1.5	µm
Δ*x*	lattice grid spacing	5	nm
Δ*t*	time increment	0.5	µs
	*lateral diffusion at the disc membrane*		
*C*_E_	density of PDE holomers in disc membrane	80	µm^−2^
*D*_R*_	lateral diffusion coefficient of R*	1.5	µm^2^ s^−1^
*D*_G*_	lateral diffusion coefficient of G*	2.2	µm^2^ s^−1^
*D*_PDE_	lateral diffusion coefficient of PDE	1.2	µm^2^ s^−1^
*D*_PDE*_	lateral diffusion coefficient of PDE*	1.0	µm^2^ s^−1^
	stochastic R* shut-off [9]		
*M*	minimum phosphates required before Arr binding	3	
*ν*, *κ*, *μ*	rate constants of R* shut-off reactions	60	s^−1^
*ρ*_low_	fractional R* activity in low-activity state	0.1	
	*rates of transducin activation and PDE shut-off*		
*ν*_RG_	rate at which fully active R* creates G*s	1000	s^−1^
*k*_E*_	rate constant of PDE* decay to PDE	2.5	s^−1^
*k*_E**_	rate constant of PDE** decay to PDE*	5	s^−1^

In the Results section, we will show that the simulations predict that a single photoisomerization causes the ensemble mean number of active PDE** molecules to begin rising approximately as a delayed ramp, and we will fit this simulated kinetics with the analytical expression2.1
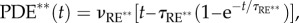
where *ν*_RE**_ is the slope of the linear ramp, and *τ*_RE**_ is the time constant of a first-order delay from the time of photoisomerization.

### 2.2. Numerical simulation of two-dimensional diffusion and molecular interactions

The numerical calculations presented here were performed using a custom program WalkMat coded in Matlab (The MathWorks, Inc., Natick, MA, USA). In addition, and as described below, checks were undertaken using an earlier program Walk2, in some restricted conditions where the two programs could simulate identical scenarios. All code used here is available for download from Dryad [10].

To estimate the number of PDEs activated during the rod's single-photon response, we simulated the reactions on a disc surface. Lateral diffusion of molecules in the plane of the disc membrane was simulated by the approach described previously [8], using a two-diemensional square-grid array with parameters appropriate for mammalian rods. As previously, the grid spacing (pixel size, Δ*x*) was 5 nm and the diffusing molecules were represented as ‘single pixels’. For computational speed, we again adopted the ‘shortcut G* production’ method [8], which enables us to ignore diffusion of the larger number of inactive G-protein molecules, and instead to simulate diffusion only of the PDEs and the active G*s and R*. Thus, as the single-active rhodopsin molecule (R*) diffuses, it activates G*s stochastically at a specified mean rate, at whatever position it occupies at the relevant instants (figure 1*b*). A consequence of this shortcut is that potential local depletion of inactive G-protein is ignored, but this simplification was previously shown to have very little effect for a single R* per disc surface [8].

Contact between the diffusing molecules was defined to occur when they occupied the same grid position (i.e. a 5 nm × 5 nm region). This differs from the previous implementation, where molecules were not permitted to occupy the same grid position and where contact was defined to occur when they were located at adjacent grid positions (at any of the four compass positions); as a result, there will be a slight difference in ‘collision radius’ between the two implementations. The disc surface was simulated as a circular region, 1.5 µm (300 pixels) in diameter. The standard density employed for the PDE holomers was 80 µm^−2^, giving 141 dimers distributed across the disc surface. The default set of diffusion coefficient and related parameters for a mammalian rod are listed in table 1.

Two approaches to simulating purely time-dependent reactions (e.g. shut-off reactions that are independent of spatial position) are possible. First, one could take each time increment and determine the probability of reaction (decay) over that time interval, and simulate whether or not the reaction occurred. Alternatively, at the instant of creation of each active molecule, one could simulate its stochastic lifetime. We adopted the latter approach, because the first approach requires more calls to the random number generator and also because it relies on the uniformity of the pseudo-random numbers over a very narrow range (e.g. with *ν*_RE_ = 5 s^−1^ and Δ*t* = 0.5 µs, the probability of PDE** decay is *ν*_RE_ Δ*t* = 2.5 × 10^−6^ in each time interval).

A second substantial shortcut in computational time was possible because of the moderate number of G* molecules created. Once the single R* had shut-off and every G* had bound (to a PDE or a PDE*), there was no longer any need to continue the simulation of lateral diffusion, because no more G*s could be created and no further ‘contact’ reactions could occur. This ‘exhaustion’ of R* and free G* typically occurred at between 50 and 200 ms of simulation time, and in practice was set by the binding of the last molecule of free G*. Thereafter the remaining reactions comprised only the GTPase-mediated shut-off of PDE** and PDE*, which involved no spatial interactions and could, therefore, be simulated very rapidly. In summary, we simulated lateral diffusion until exhaustion of R* and free G* occurred, and then only the time-dependent reactions until all other molecules of interest (PDE* and PDE**) had decayed.

#### 2.2.1. Check on numerical simulations

As a check on our numerical simulation algorithm, we were able to make a comparison with the predictions of a completely independent program, Walk2, that had been written in 1996 by Lucian Wischik. That program was coded in C++ and is specific to the Windows operating system. It has the great advantage of executing very rapidly, though in part this is because it uses a ‘quick-and-dirty’ random number generator, whereas Matlab uses the more computationally demanding Mersenne twister algorithm [11]. On the other hand, Walk2 has some limitations that prevent its use in our main calculations. For example, it is restricted to a square (rather than circular) region of the disc, but more importantly, it cannot implement multi-stage shut-off of R* of the kind required by multiple phosphorylations and arrestin binding.

Nevertheless, there were some restricted conditions under which we could compare the predictions of the two programs. We chose the case of a disc surface with: square geometry, of width 1.34 µm (to maintain the area); a single R* positioned randomly at time zero; stochastic R* decay with a single rate constant (15 s^−1^); ‘shortcut’ generation of G*; dimeric PDE; stochastic PDE* and PDE** decay; diffusion-limited reactions; 1000 trials. With the exception of the geometry and the single-stage R* decay, all parameters were set to the default mammalian values listed in table 1. The results for Walk2 were qualitatively very similar to those for WalkMat, though the trace for the mean PDE** time-course was marginally larger; when the Walk2 PDE** trace was scaled vertically by a factor of 0.86 it was virtually indistinguishable from the WalkMat PDE** trace (data not shown). We think that this difference arose primarily because of the smaller effective collision radius in the WalkMat implementation. Overall, though, the similarity of the responses gives us confidence that the WalkMat program is likely to be generating meaningful predictions.

### 2.3. Downstream reactions: model and numerical integration

The experimentally measured response of a rod photoreceptor is its electrical activity, typically recorded as the circulating current or the intracellular voltage, and this electrical response is the result of PDE activity (either light-induced or spontaneous) acting via the downstream phototransduction cascade. We simulated these downstream reactions using the same equations as presented recently [9], except with *β*_sub_ replaced by *β*_E**_. Thus, we used a conventional description of the downstream reactions, including Ca^2+^-mediated feedback via GCAPs, in conjunction with longitudinal diffusion of cGMP and Ca^2+^ within the cytoplasm. The full set of equations was given in the section ‘Downstream phototransduction cascade’ on p. 680 in [9], and is included here as electronic supplementary material. Numerical integration of the partial differential equations was based on the earlier program RodSim [12], and the code used here is included in the WalkMat package [10].

Using the parameters in the top section of table 2, we calculated the dark resting state by setting all-time derivatives to zero, as previously described (p. 683 of [9]), and the resulting steady-state values are listed in the second section of table 2. For our later determination of the hydrolytic activity required to reduce the circulating current by 90%, we used a similar approach. We again set all-time derivatives to zero, and then evaluated all variables over a wide range of Ca^2+^ concentrations, thereby enabling us to relate steady hydrolytic activity (*β*) to steady circulating current (*J*).

**Table 2.** Downstream phototransduction cascade parameters. The driving function for the downstream reactions was a specified PDE**(*t*) time-course at the spatial element midway along the length of the outer segment. Radial diffusion in the cytoplasm is very rapid and is ignored in this model, so that the radial location on the disc membrane of any PDE** is irrelevant. For the semi-discretization method of simulating the longitudinal diffusion of cGMP and Ca^2+^, the outer segment was divided into 51 compartments, with the two end compartments (at *x* = 0 and *x* = *L*) being half the width of the remainder.

symbol	description	value	units
*β*_Dark_	dark rate constant of cGMP hydrolysis	4.0	s^−1^
*α*_max_	maximal rate of cGMP synthesis by GC	150	µM s^−1^
*f*_Ca_	fraction of CNGC current carried by Ca^2+^	0.12	
*K*_GCAP_	Ca^2+^ concentration parameter of GCAP	80	nM
*m*_GCAP_	Ca^2+^ cooperativity of GCAP	1.5	
*J*_cG, max_	maximal CNGC current for the OS	2000	pA
*n*_cG_	cooperativity of CNGC activation by cGMP	3	
*K*_cG_	cGMP concentration parameter of CNGCs	20	µM
*J*_ex, max_	maximal exchange current for the OS	4.6	pA
*K*_ex_	Ca^2+^ concentration parameter of exchanger	1100	nM
	*calculated resting dark state*		
cG_Dark_	dark cGMP concentration	4.12	µM
Ca_Dark_	dark Ca^2+^ concentration	322	nM
*α*_Dark_	dark rate of cGMP synthesis by GC	16.5	µM s^−1^
*J*_Dark_	dark current	18.4	pA
	*parameters not affecting the resting state*		
*β*_E**_	rate constant of cGMP hydrolysis by a PDE**	0.025	s^−1^
*V*_cyto_	available cytoplasmic volume of OS	0.02	pL
*B*_Ca_	buffering power of cytoplasm for Ca^2+^	50	
	*longitudinal diffusion parameters*		
*D*_cG_	longitudinal diffusion coefficient for cG	40	µm^2^ s^−1^
*D*_Ca_	longitudinal diffusion coefficient for Ca^2+^	2	µm^2^ s^−1^
*L*	length of outer segment	22	µm

To predict the electrical responses to single photoisomerizations, we used a large set of simulated PDE**(*t*) waveforms as driving functions for the downstream reactions described above, with the parameters listed in table 2. For the GCAPs^−/−^ case, we simply held the guanylyl cyclase rate *α*(*x*, *t*) at the level *α*_Dark_ determined in the dark resting state for WT rods.

To predict the unitary electrical responses elicited by spontaneously created PDE**s, we simulated the individual PDE** events and used these as driving functions for the same downstream reaction equations; this case is much more straightforward to simulate because we do not need to consider lateral diffusional interactions at the disc membrane. The PDE** events were either: (*a*) a set of 1000 rectangular events of unit amplitude, each starting at time zero and having a stochastic duration that was exponentially distributed with time constant *τ*; or (*b*) a single exponential decay starting from unity at time zero and having the same time constant, *τ*.

### 2.4. Amplification of the single-photon response

According to the accepted quantitative model of the vertebrate phototransduction cascade [1,13], when the hydrolytic activity of the PDE rises as a ramp with time, then the rising phase of the photoreceptor's electrical response to a brief flash of light can be described approximately as a ‘delayed Gaussian’ function2.2
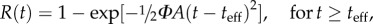
where *R*(*t*) is the fractional response, *Φ* is the flash intensity (in photoisomerizations), *A* is the amplification constant (in s^−2^) characterizing the cell, and *t*_eff_ is a short ‘effective delay time’. This description applies only at times sufficiently early that shut-off reactions have not contributed appreciably. Equation (2.2) has been shown to provide a good description of the early rising phase of the electrical response to flashes, in many studies, and for rods from a wide variety of species (reviewed in [13]). For rodent rods, the estimates of amplification constant *A* that have been reported in the literature range from 5 to 23 s^−2^ [14–18]. It is likely that some of the lower values resulted from excessive low-pass filtering [17], and we recently suggested that the true value in WT mouse rods is likely to be *A* ≈ 24 s^−2^ [9].

The standard quantitative model for the response rising phase provides an expression for the amplification constant *A* in terms of biochemical and physical parameters [1,13]. However, that expression was derived for independently activated subunits of PDE* so that, in terms of our present model of PDE**, the expression needs to be rewritten as2.3
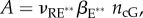
where *ν*_RE**_ is the rate at which a single active R* triggers activation of PDE** catalytic holomers, *β*_E**_ is the hydrolytic efficacy of a fully activated PDE**, and *n*_cG_ is the cooperativity of cGMP-gated channel opening by cGMP. As in the original study, it is possible to express *β*_E**_ in terms of more basic physical and biochemical parameters, as2.4
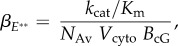
where *k*_cat_ and *K*_m_ are the catalytic activity and Michaelis constant for the fully activated PDE** holomers, *N*_Av_ is Avogadro's number, *V*_cyto_ is the cytoplasmic volume of the outer segment, and *B*_cG_ is the cytoplasmic buffering power for cGMP. As a result, equation (2.3) can be rewritten as2.5
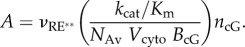


### 2.5. Power spectral density of stochastic events

In order to predict the power spectrum of the noise elicited by spontaneous occurrences of ‘unitary’ events, we first calculated the one-sided power spectral density, *S*(*f*), of the simulated events, using the fast Fourier transform implemented in the ‘fft’ function in Matlab. The one-sided spectrum is defined only for positive frequencies, and so is double the two-sided spectrum. In this case, the variance of the original signal should be equal to the integral of its spectrum over positive frequencies2.6
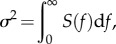
which we confirmed in our numerical analysis by summing the spectral densities and multiplying by the frequency interval.

For identical events *r*(*t*), occurring stochastically in time at a mean rate *ν* s^−1^, the zero-frequency asymptote of the one-sided spectral density is predicted to be2.7
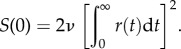
If these identical events have an exponential decay from unit amplitude, *r*(*t*) = exp(−*t*/*τ*), then the predicted spectral density is the well-known Lorentzian function2.8
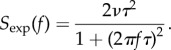
When instead the individual events are not identical, but have unit amplitude and stochastic lifetime *τ*, so that the ensemble mean is unchanged at exp(−*t*/*τ*), the resulting power spectral density has exactly the same shape but is simply scaled vertically by a factor of 2, as2.9
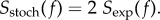
This last result was derived in 1976 by Prof. Peter Whittle of the Statistical Laboratory, University of Cambridge, for theoretical analysis in a study of cone photoreceptor noise [19].

### 2.6. Responses to bright flashes

For calculating the responses to bright flashes, where multiple disc surfaces receive isomerizations, we used a ‘single compartment’ model of the downstream reactions. Furthermore, we allowed for the fact that individual disc surfaces may receive multiple isomerizations. Thus, we simulated the time-course of PDE**(*t*) activity in a single disc surface for multiple isomerizations, in the case of 2, 3, … 10 isomerizations, and also for 20 and 30 isomerizations (though the last two cases were very time-consuming). As described in the Results, we developed an approximate approach for estimating the PDE**(*t*) time-course for other integer numbers of isomerizations per disc surface, below 30. Then, for each integer *k* = 0 … 30, we calculated the probability *p_k_* that *k* photoisomerizations would occur in any disc surface, as follows. When the mean number of photoisomerizations per disc surface is *φ* (and hence the number of photoisomerizations per outer segment is *Φ* = *N*_surf_ *φ*), the Poisson distribution gives *p_k_* as2.10
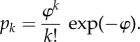


Therefore, to calculate the electrical response, we summed the PDE**(*t*) predictions described above, after weighing them according to the proportion of surfaces that would have absorbed that number of isomerizations, and we used the resulting weighted PDE**(*t*) time-course as the driving function for the downstream reactions.

Our use of the shortcut of a constant mean rate of G* production approach places a restriction on the upper intensity we can investigate, in order to avoid depletion of transducin. For example, a flash of *Φ* = 25 000 isomerizations per rod (a 0.06% bleach) delivers an average of *φ* = 17 isomerizations per disc surface. In conjunction with an R* mean lifetime of approximately 70 ms and a shortcut G* activation rate of 1000 s^−1^, this would create 1200 G*s, representing about 22% of the complement of 5300 transducins per disc surface. As a result, our neglect of transducin depletion should have little effect below 10 000 isomerizations per rod, but at levels of 25 000 isomerizations or more it is likely to lead to overestimation of G* production, and hence overestimation of the time spent in saturation.

## 3. Results

### 3.1. Predicted PDE kinetics for a mammalian rod

**Figure 2.**
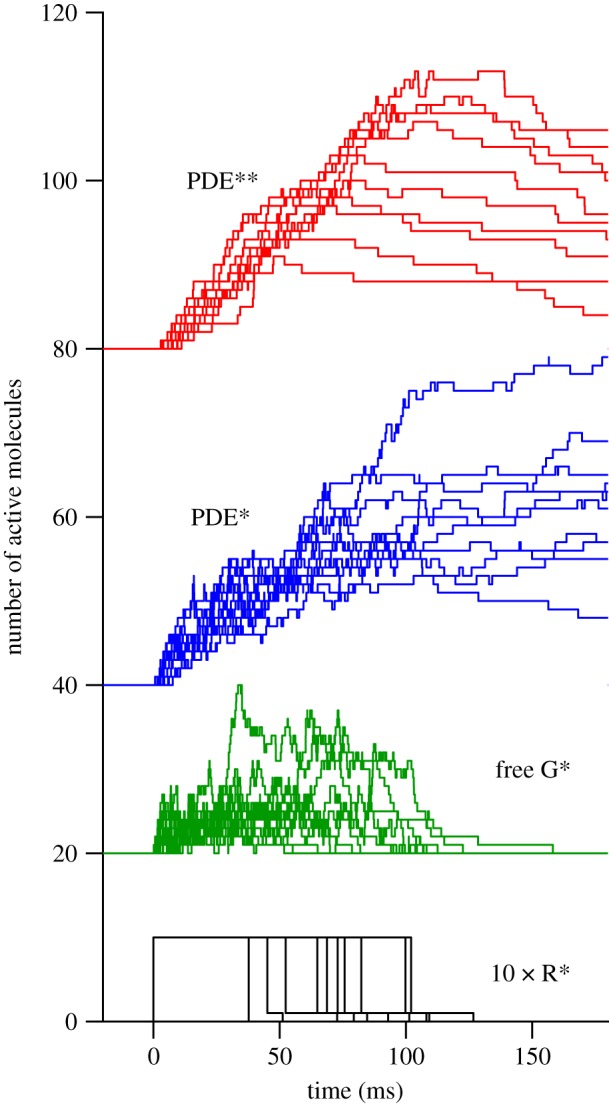
Sample raw traces for the numbers of activated molecules of different species, predicted in simulations with the standard parameters in table 1. These are the first 10 stochastic simulations from the run used to calculate the traces in the next two figures; the groups have been displaced vertically for clarity. Black traces: R* (scaled vertically 10×); the late lower level shows the low-activity state invoked by Lamb & Kraft [9]. Green traces: free G* (i.e. the number of G*s not bound to PDE). Blue traces, PDE*. Red traces, PDE**. In each case, the traces plot the numbers of molecules active, as distinct from the total numbers produced.

**Figure 3.**
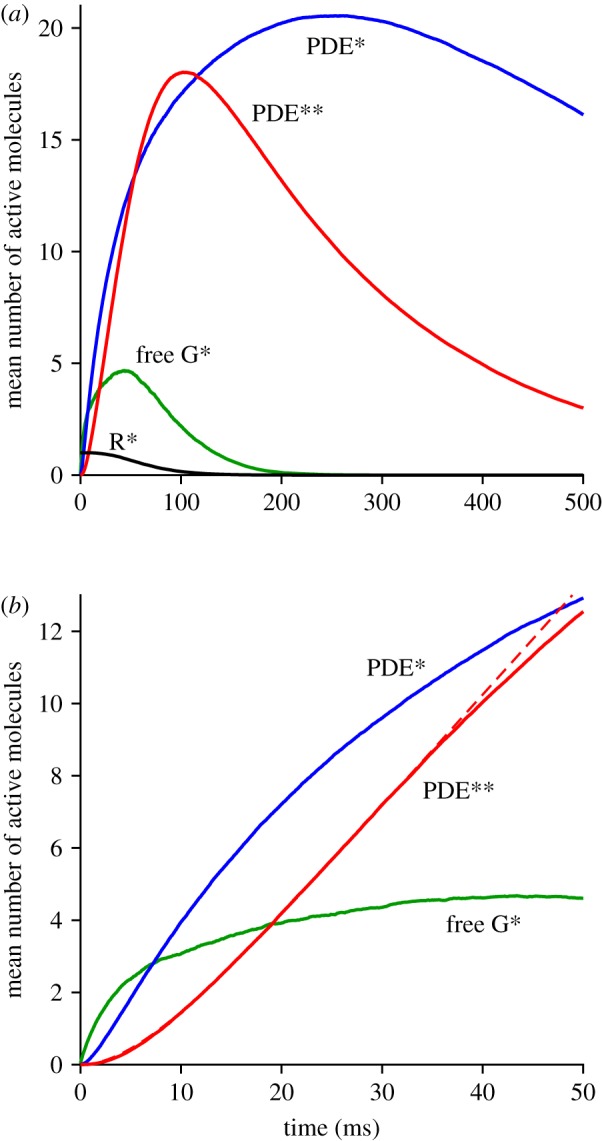
Simulated mean time-course of different species of activated molecule, for standard parameters in table 1. (*a*) Mean response over 500 ms. (*b*) Expanded time-base for the first 50 ms. Dashed red trace plots equation (2.1), fitted over the first 35 ms, yielding *ν*_RE_ = 309 PDE** s^−1^ and *τ*_RE_ = 6.9 ms.

A small set of sample traces showing the simulated numbers of molecules existing in the different states, in response to a single photoisomerization in each trial, is plotted in figure 2. Note the substantial variability that results from the stochastic nature of the underlying reactions. For 4000 repetitions, using our standard set of parameters for a mammalian rod (including shut-off steps), the ensemble mean traces are plotted in figure 3. Figure 3*a* shows a time-base to 500 ms, with traces for R* (black), free G* (green), PDE* (blue) and PDE** (red). Note that each trace plots the mean number of molecules remaining present at any instant, rather than the total created, and that for G* we exclude those molecules that are bound to PDE. For the fully activated PDE**, the ensemble mean reaches a peak of 18.0 PDE** at 102 ms after the photoisomerization. At that time, the total number of G*s to have been produced averaged 68, and of those 55 remained active (as 18 doubly bound PDE**s, 17 singly bound PDE*s and 2 free G*s). The production of 68 G*s (on average) corresponds to our simulated activation rate of 1000 G* s^−1^ times the mean R* lifetime of 68 ms, and is considerably higher than anticipated from biochemical experiments on the incorporation of GTP*γ*S [20]; this discrepancy will be discussed subsequently.

Figure 3*b* concentrates on the onset phase of the simulated responses, over the first 50 ms; it omits the R* trace, which declined only slightly from its initial value of unity. The earliest rise of the PDE** trace is roughly parabolic, and up until about 35 ms the rising phase is well described by the dashed red curve, which plots the delayed ramp expression given above in equation (2.1), with fitted slope *ν*_RE**_ = 309 PDE** s^−1^ and first-order delay *τ*_RE**_ = 6.9 ms. Note that our parameter *ν*_RE**_ refers to doubly bound PDE**s rather than to individual PDE subunits.

These results reveal several features of the PDE6 dimeric activation model, in the case of the predicted response of a mammalian rod to a single photoisomerization. First, at early times, prior to any significant shut-off (i.e. before approx. 35 ms), the time-course of PDE** activity rises as a delayed ramp (i.e. the convolution of a linear ramp with an exponential decay, as defined in equation (2.1)). Apart from this delay, such a ramp is the standard behaviour predicted by the conventional molecular model of phototransduction that assumes independent PDE catalytic subunits [1]. Second, the addition of a time constant of approximately 5–7 ms for a rod at mammalian body temperature is just what is needed to explain the recent discovery by Rotov *et al.* [7] of a ‘paradoxical’ delay of approximately 10 ms in experiments on frog rods at room temperature. Third, the slope of the ramp, at 309 PDE** s^−1^, is only 62% of the maximum possible rate, of 500 PDE** s^−1^ (for a G* activation rate of 1000 G* s^−1^). We refer to this ratio as the efficacy *η*_GE**_ of PDE** activation by G*, defined as *η*_GE**_ = 2 *ν*_RE**_/*ν*_RG_ (where the factor of 2 is required because each PDE** formed has two G*s bound). This coupling efficacy is considerably smaller than unity because a substantial proportion of the total G*s is accounted for by the singly bound form, PDE*. Fourth, the green trace for free G* in figure 3*b* shows that, on average, only a handful of G* molecules are available to bind at any moment. Hence, although the efficacy of G* in creating PDE** is relatively low, its efficacy in binding to *some* configuration of the PDE is very high.

### 3.2. Predicted electrical response to a single photoisomerization

**Figure 4.**
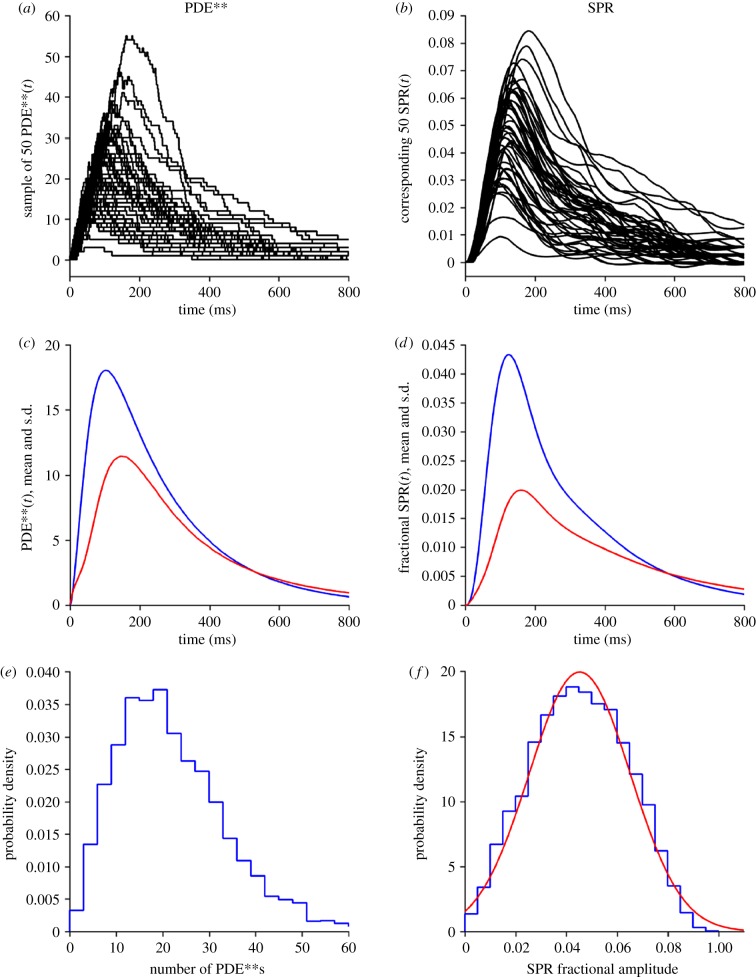
Single-photon PDE** kinetics and electrical response. Results for simulation of 4000 trials with the standard parameters listed in table 1. Column (*a,c,e*) shows PDE**; column (*b,d,f*) shows electrical response (normalized, as a fraction of the circulating dark current). Row (*a,b*) shows a sample of 50 consecutive simulations. Row (*c,d*) shows the ensemble means (blue) and s.d. (red). Row (*e,f*) shows the probability density histograms (blue); the red curve in panel (*f*) is a Gaussian with mean of 0.0453 and an s.d. of 0.0200.

We next determined the predicted electrical response to a single photoisomerization, by solving the differential equations for the downstream reaction cascade when driven by the simulated kinetics of PDE**. Figure 4*a* shows a sample of 50 simulated traces for PDE**, and figure 4*b* shows the corresponding predicted electrical responses for the same set of simulations. At late times in figure 4*b*, it is possible to discern the electrical response to the last few remaining molecules of PDE**. For the 4000 trials, the ensemble means (blue) and standard deviations (red) are shown for PDE** in figure 4c, and for the electrical response in panel *d*. The ensemble mean single-photon response (SPR) has a peak of 0.0432 (i.e. 4.3% of the dark current). This peak occurs at 125 ms, and the delay of 23 ms from the PDE** peak at 102 ms reflects the filtering properties of the downstream reaction cascade. This filtering results from an integrating stage, with a time constant set by the cGMP turnover time, 1/*β*_Dark_, due to resting PDE activity, in conjunction with the action of the Ca^2+^ feedback loop.

The plots of ensemble standard deviation in figure 4*c* and *d* provide a representation of the variability of the simulated SPRs, and, as has been reported in experiments on mammalian rod SPRs, the standard deviation (or its square, the variance) peaks later than does the ensemble mean [21]. In order to provide a more informative measure of response variability, that study proposed an analysis of the SPR area (i.e. its time integral), and in particular, its coefficient of variation (ratio of s.d./mean), denoted here as CV_area_ [21]. Subsequently, a theoretical basis for the importance of using CV_area_ was developed [22], with the interpretation that under suitable conditions, the minimum number of stages involved in R* shut-off would be 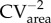. For the simulations in figure 4, we calculated CV_area_ = 0.671 for PDE** and CV_area_ = 0.561 for the electrical responses. These CVs are larger than the values of up to 0.35 reported for mammalian rod SPRs [21,22], and presumably reflect the combination of our choices, first, of the number of phosphorylation steps in R* shut-off as being three and, second, parameters yielding only a moderate number of PDE**s during the SPR.

Finally, the bottom row of figure 4 plots histograms for the probability density of the maximum amplitudes measured from the individual simulations. For PDE**, the distribution in figure 4*e* is quite asymmetric, with a pronounced tail of large peaks (consistent with the occurrence of a few very large traces in figure 4*a*). For the SPRs, the distribution in figure 4*f* is more nearly symmetrical. The red curve plots a Gaussian distribution with the measured distribution mean of 0.0453 and with a coefficient of variation for the amplitudes of CV_ampl_ = 0.44, which is somewhat higher than typically reported in electrical recordings of mouse rod SPRs.

### 3.3. Dependence of PDE** activation on the rate of transducin activation

**Figure 5.**
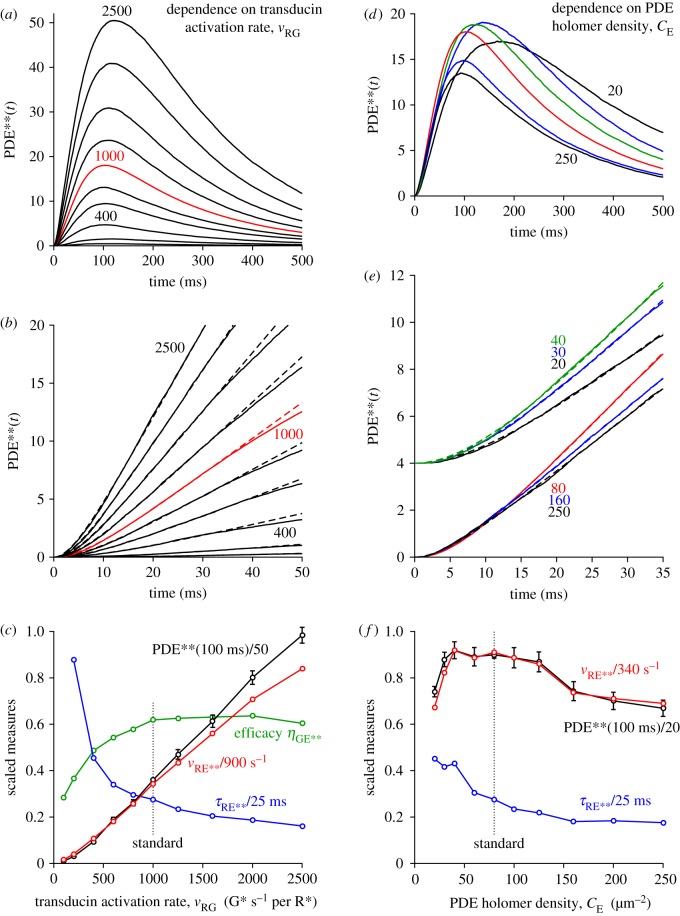
Effect of altered rate of G* activation (*a–c*) and altered PDE density (*d–f*) on simulated PDE** responses. (*a,d*) Simulated responses on a time-base of 500 ms. (*b,e*) The same responses on an expanded time-base, with equation (2.1) fitted over the interval 0–35 ms (dashed traces). (*c,f*) Measurements extracted from the panels above: PDE** at a fixed time near the peak (black); the fitted slope of E** activation (red); initial delay (blue); and in (*c*), efficacy *η*_GE**_ of E** activation (see text). In order to show multiple parameters in a single panel, the measurements have been scaled vertically as indicated. The error bars for the measured PDE** levels (black) show the 95% confidence intervals, calculated as ±2 s.e.m. The dotted vertical lines indicate the standard parameter values: a G* activation rate of 1000 s^−1^ in (*c*), and a PDE density of 80 holomers µm^−2^ in (*f*); all other parameters were held constant at their standard values listed in table 1. For (*a–c*), the G* activation rates were: *ν*_RG_ = 100, 200, 400, 500, 800, 1000, 1250, 1600, 2000, 2500 G* s^−1^. For (*d–f*), the PDE densities were: *C*_E_ = 20, 30, 40, 60, 80, 100, 125, 160, 200, 250 holomers µm^−2^ (though only a subset of time-course traces are plotted, as indicated in (*e*)); the three traces for the lowest PDE densities in (*e*) have been offset vertically to avoid overlap. The traces in the upper four panels are the means for 500 simulations, except for the red traces using the standard set of parameters which are taken from figure 3 with 4000 simulations.

The rate of transducin activation adopted here, of *ν*_RG_ = 1000 G* s^−1^, is higher than the value of 300 G* s^−1^ that has recently been assumed for mammalian rods [9,23,24] (based on extrapolation from the report of 120–150 G* s^−1^ for amphibian rods at room temperature [20]), but it conforms with estimates obtained using light-scattering measurements [25–28]. In the next set of calculations, we altered this rate from 100 to 2500 G* s^−1^ while holding all other parameters constant. Figure 5*a–c* presents the results of these simulations, averaged across at least 500 trials in each case. Figure 5*a* plots the ensemble means of the simulated responses, and it is clear that the activation of PDE** depends strongly on the rate of G* activation. For example, in the third trace (from the bottom), where *ν*_RG_ was reduced to 400 G* s^−1^ (still higher than the level assumed in recent modelling studies), the mean number of PDE** molecules per trial peaked at only 4.7, roughly a quarter of the peak obtained with our standard rate of 1000 G* s^−1^. This demonstrates that, in order for the PDE dimeric activation model to have a chance of providing a plausible description of the rod's response, it is necessary that the rate of G* activation be higher than has been assumed in previous modelling of rod phototransduction. Figure 5*b* plots these same traces on an expanded time-base, and (as for figure 3) the dashed curves plot equation (2.1), in each case providing a good description of the early rising phase.

In figure 5*c*, we plot several measures derived from these simulated PDE** responses against the rate of G* activation, and we indicate the standard rate of G* activation with the dotted vertical line. The response amplitudes, measured from figure 5*a* at a fixed time near the peak, of 100 ms, are plotted as the black symbols, and have been scaled to a maximum of 50 PDE**. The fitted slopes (*ν*_RE**_) are plotted as the red symbols, scaled to 900 PDE** s^−1^. The fitted time constants (*τ*_RE**_) are plotted as the blue symbols, scaled to 25 ms. Finally, the efficacy (*η*_GE**_) of PDE** activation by G* is plotted in green. Clearly, there is a powerful dependence of the amplitude and the fitted slope on the rate of G* activation used in the simulations. And because of the curvature of this relation at low rates of activation, the calculated efficacy of coupling increases as a function of G* activation rate, before approaching a plateau. On the other hand, the fitted time constant declines with increasing G* activation rate. For the two lowest rates of G* activation (100 and 200 G* s^−1^), the responses were so small that the fitted parameters could not be relied upon; the time constant at the lowest rate was greater than 25 ms and has been omitted from the plot.

### 3.4. Dependence of PDE** activation on the membrane density of PDE

We next investigated the effect of altering the membrane density of PDE6 holomers on the simulated activation of PDE**, and found a relatively weak dependence, as shown in the right-hand column of figure 5. Figure 5*d* shows a superimposed plot of ensemble mean responses for six representative membrane densities, of *C*_E_ = 20, 30, 40, 80, 160 and 250 holomers µm^−2^; all other parameters (including the rate of G* activation) were held constant at their standard values. The rising phase of each of these traces is plotted in figure 5*e*, and because of the overlapping nature of the superimposed traces in figure 5*d*, we have shifted three of them vertically for clarity; the upper group plots the lowest three densities, and the lower group plots the highest three densities. Again, the dashed traces plot the predictions of equation (2.1), and in each case, the fit is very good.

Measurements from these responses are plotted against PDE membrane density in figure 5*f*, in a corresponding manner to figure 5*c*, and again in scaled form. In this case, both the amplitude measured at 100 ms (black) and the fitted rate of rise *ν*_RE**_ (red) decline on either side of a broad peak that occurs at between about *C*_E_ = 40 and 80 PDE holomers µm^−2^. The fitted time constant of delay *τ*_RE**_ (blue) decreased with increasing density of PDE. (We have not included a trace for the efficacy *η*_GE_ of PDE** activation, as this had exactly the same shape as the red trace for *ν*_RE_ because the transducin activation rate *ν*_RG_ was fixed.)

It is natural to expect the delay time to decrease with increasing PDE density, but the decline in slope and amplitude at higher densities is less intuitively obvious. We interpret those declines to indicate that, at higher densities of PDE, the fractional level of singly bound PDE* (relative to unbound PDE) at any instant is lower than it is at moderate PDE densities, so that each newly created G* has a lower probability of finding a PDE* than it would have at a moderate membrane density of PDE. We suspect that this phenomenon is closely related to the biochemical observation (in fig. 1*c* of [4]) that at high membrane densities of PDE there is a reduction in the proportion of doubly bound PDE** that is formed.

### 3.5. Unitary electrical responses to individual PDE** activations and the continuous noise

We next investigated the consequences that dimeric activation of PDE has for the magnitude and properties of the spontaneous ‘continuous’ component electrical noise in rod photoreceptors. To do this, we calculated the electrical response elicited by the spontaneous activation of an individual PDE** molecule, and we calculated the noise spectrum predicted for such events occurring stochastically in time (see §2.5). For comparison with experimental recordings reported in the literature, where the ‘continuous’ noise is much greater in the rods of GCAPs knockout mice than in the rods of WT mice, we calculated the responses and power spectra for both genotypes. For the GCAPs^−/−^ case, we simply held the guanylyl cyclase activity fixed at the dark resting level obtained for WT rods.

Spontaneous activation of PDE** would be expected to result from the binding of a spontaneously activated G* to a molecule of singly bound PDE*. The resulting PDE** would be expected to remain present until inactivated stochastically as a result of GTPase activity occurring with a rate constant *k*_E**_ (taken to be 5 s^−1^). We shall refer to this spontaneous occurrence as a ‘unitary PDE** event’; its ensemble mean (the mean unitary PDE** event, *û*(*t*)) will be an exponential decay from unity, *û*(*t*) = exp(−*k*_E**_ *t*). We therefore solved the downstream reactions for two scenarios of driving functions: (*a*) a large set of rectangular PDE** events with stochastic lifetimes that were exponentially distributed with a time constant 1/*k*_E**_; and (*b*) a single event in the form of an exponential decay with the same time constant. The ensemble mean of the electrical responses for (*a*) should be identical to the single electrical response for (*b*), provided that the downstream reactions behave linearly for such small perturbations. But, interestingly, for events of these types occurring randomly in time, the power spectral density is predicted to be scaled vertically by a factor of 2 in the former case, in comparison with the case for random occurrences of identical exponential events. This result was derived in 1976 by Prof. Peter Whittle of the Statistical Laboratory, University of Cambridge; see §2.5.

**Figure 6.**
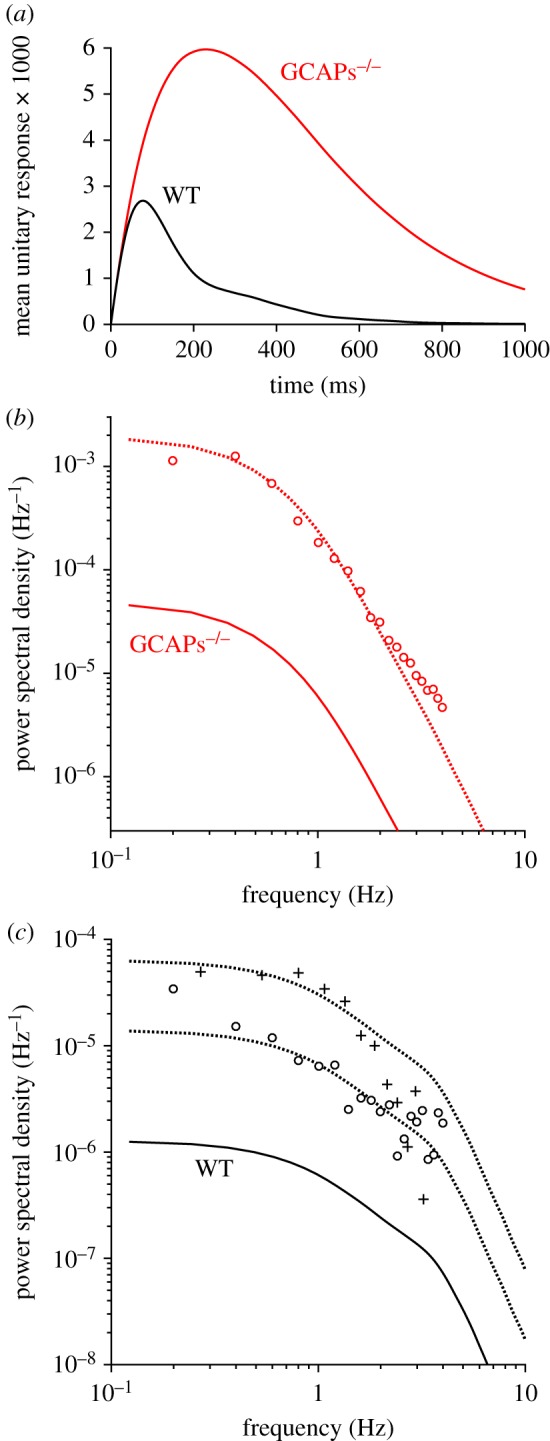
Unitary PDE** event electrical responses and power spectral density. (*a*) Mean electrical responses to activation of a single PDE**, averaged from 1000 simulations for individual events with exponentially distributed lifetimes. Trace WT (black) is for wild-type mammalian rods, using the standard parameters in table 1; trace GCAPs^−/−^ (red) is for GCAPs knockout, modelled by holding the guanylyl cyclase rate constant at the resting level for WT rods. (*b*) One-sided power spectral density, for GCAPs^−/−^ rods. Solid red trace was predicted by averaging the spectra of the individual simulated GCAPs^−/−^ responses used to construct panel (*a*), and corresponds to a mean rate of stochastic events of 1 s^−1^. Symbols plot GCAPs^−/−^ rod data from fig. 4*b* of Burns *et al.* [29], normalized for their circulating current of 14.0 pA. Dotted red trace is scaled vertically from solid red trace using an event rate of 40 s^−1^. (*c*) One-sided power spectral density, for WT rods. Solid black trace is predicted from the simulated WT responses used in panel (*a*). Open symbols plot WT data from fig. 4*b* of Burns *et al.* [29], normalized for their circulating current of 12.9 pA; adjacent dotted trace is scaled for an event rate of 11 s^−1^. Symbols ‘+’ plot data for WT monkey rods from fig. 14*b* of Baylor *et al.* [30], normalized for their circulating current of 13 pA; adjacent dotted trace is scaled for an event rate of 50 s^−1^.

We solved the differential equations for the downstream reaction cascade in response to a set of 1000 simulated unitary PDE** events having unit amplitude and exponentially distributed lifetimes, and the predicted mean unitary PDE** electrical response is plotted (as a fraction of the dark current) in figure 6*a*, as the black trace for WT, and as the red trace for GCAPs^−/−^. The two unitary responses rise without delay, along a common initial time-course. The WT trace reaches a peak amplitude of 2.7 × 10^−3^ (i.e. 0.27% of the dark current) at 77 ms; this peak is 1/16 that of the mean single-photon electrical response (0.0432) in figure 4*d*, broadly as expected given that a mean of 18 PDE**s are present at the peak of the mean simulated WT single-photon response (figure 4*c*). The GCAPs^−/−^ trace reaches a much larger peak amplitude of 6.0 × 10^−3^ at 229 ms. As a check, we calculated the response of the downstream cascade when the driving function was the ensemble mean unitary PDE** event, *û*(*t*) = exp(−*k*_E**_ *t*), and for both genotypes the response was almost identical to the illustrated traces (not shown), consistent with the idea that, for stimuli as small as a single PDE**, the downstream reactions operate effectively as a linear filter. Thus, for both genotypes, one can think of the traces in figure 6*a* as representing the convolution of an exponential decay (having a 200 ms time constant) with the small-signal transfer function of the downstream reactions for the genotype; this generates a peak at 77 ms for WT, and at 229 ms for GCAPs^−/−^.

In figure 6*b*,*c*, we have plotted as the solid traces the power spectral density averaged from the individual downstream responses to the 1000 stochastic unitary PDE** events, for GCAPs^−/−^ and WT genotypes, respectively. For these solid traces, the vertical scaling corresponds to a stochastic event rate of *ν* = 1 s^−1^ (see §2.5). As a check, we also calculated the power spectral density for the mean unitary electrical response (for each genotype), and confirmed that upon vertical scaling by a factor of 2 this was closely similar to the illustrated spectrum (not shown). In these log–log coordinates, the power spectral density at high frequencies declines with a slope of −4 for both genotypes, as expected with two ‘integrating’ stages, representing the mean PDE** lifetime, 1/*k*_E**_, and the cGMP turnover time, 1/*β*_Dark_, in the downstream cascade.

To compare our predicted spectra with spectra reported in the literature for the continuous noise recorded from mammalian rods, we began with recordings from GCAPs knockout mice, where the noise is substantially larger. Burns *et al.* [29] have presented power spectral density analysis of the dark noise from rods of both WT and GCAPs^−/−^ mice in their fig. 4b. Although they did not separate the ‘continuous’ component of noise from the effects of spontaneous photon-like events, inspection of their fig. 3 suggests that their recordings were dominated by the continuous component. Because all of our analysis is in terms of normalized response (response divided by circulating dark current), we normalized their power scale by dividing by the square of the rod dark current (GCAPs^−/−^ 14.0 pA, WT 12.3 pA). The resulting values from fig. 4*b* of [29], for measurements up to 4 Hz, are plotted as the open symbols in figure 6*b*,*c*. The dotted traces near these open symbols plot the corresponding solid traces scaled vertically by assumed rates of stochastic PDE** events of 40 s^−1^ (GCAPs^−/−^, figure 6*b*) and 11 s^−1^ (WT, figure 6*c*). In addition, in figure 6*c*, we have plotted as the ‘+’ symbols the measured spectra for WT monkey rods taken from fig. 14*b* of Baylor *et al.* [30] and normalized for their dark current of 13 pA. In this case, the adjacent dotted trace plots the solid trace scaled vertically by an assumed rate of stochastic PDE** events of 50 s^−1^.

Thus, the rate of stochastic PDE** events used for the three dotted traces were: mouse GCAPs^−/−^, 40 s^−1^; mouse WT, 11 s^−1^; monkey WT, 50 s^−1^. Overall, we think that these fits are as good as can be expected, given the difficulties that potentially result from very slow drift (causing problems at the lowest frequencies) and the occasional occurrence of spontaneous photon-like events. The required vertical scaling for the three sets of data from the literature suggests that stochastic activation of the PDE** occurs at a rate of the order of a few tens per second, and we shall address the interpretation of this estimate in the Discussion.

### 3.6. Responses to multiple photoisomerizations per disc surface

Up to this point, we have considered only the very smallest responses (i.e. responses to single photoisomerizations per rod, and the even smaller responses to activation of individual PDE** molecules). As the flash intensity delivered to a rod increases from the lowest levels, the rod's response initially scales in direct proportion to flash intensity, with negligible change in time-course; this is termed the ‘linear range’ and typically applies for responses up to about 20% of maximal. At higher intensities, the peak initially moves slightly earlier, in a manifestation of light adaptation. When the response amplitude is measured at a fixed time, before this peak, the relationship between response amplitude (*R*) and flash intensity (*Φ*) saturates according to an exponential function, *R*/*R*_max_ = 1 – exp(−*kΦ*) [1,31]. Saturation is typically reached at an intensity of a few hundred photoisomerizations, but as these isomerizations are distributed randomly across around 1500 disc surfaces, the vast majority of disc surfaces receive either no isomerization or a single isomerization. However, at intensities high enough to hold the rod's response in saturation, multiple photoisomerizations can occur per disc, and we now investigate the PDE** activity that occurs under these conditions.

**Figure 7.**
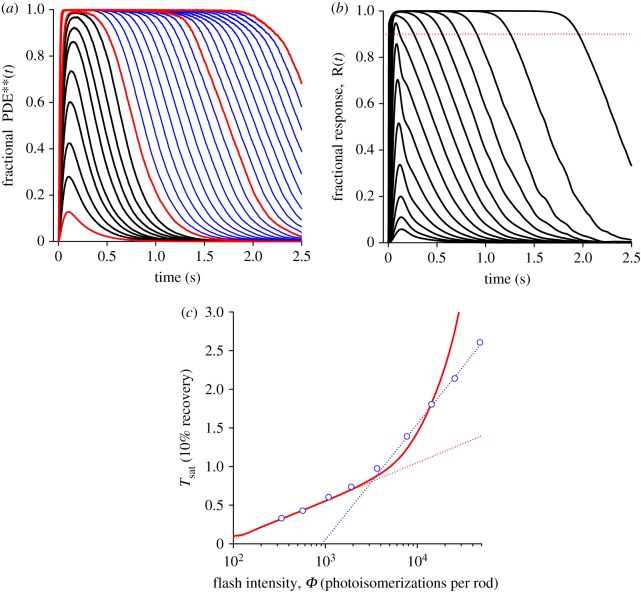
Bright flash responses. (*a*) Predicted fractional PDE**(*t*) in response to integer numbers, *φ*, of photoisomerizations per disc surface. The red and black traces plot ensemble mean responses for simulations: the red traces are for *φ* = 1, 10, 20 and 30, and black traces are for *φ* = 2 … 9 isomerizations per surface. The blue traces correspond to *φ* = 11 … 19 and *φ* = 21 … 29, and have been derived by shifting the saturated red traces by multiples of the mean observed shift of 94.1 ms and weighting them appropriately. (*b*) Predicted fractional electrical responses *R*(*t*) to flashes delivering from *Φ* = 1 to 16 000 photoisomerizations per outer segment, in steps of 0.3 log_10_ units (i.e. approximately doubling the intensity between each trace). The minor wobbles at late times in the traces at around 4000 isomerizations presumably resulted from stochastic fluctuations in the relatively small number of PDE** molecules at these times, for simulations with around 3 isomerizations per disc surface. The dotted horizontal line indicates 90% suppression of the dark current. (*c*) Time spent in saturation plotted as a function of flash intensity, *Φ* photoisomerizations per rod, plotted logarithmically. The continuous red trace is taken from predicted traces such as those in panel (*b*), but calculated at intensity intervals of 0.05 log_10_ units. The dotted red line, which approximates the predicted saturation time for flashes up to 3000 isomerizations per rod, has a slope corresponding to a dominant time constant of 215 ms. The symbols are experimental measurements for WT mouse rods taken from fig. 4 of [32], without any shifts. The dotted blue line is positioned to describe the points at the highest intensities, and its slope corresponds to a time constant of 650 ms.

Figure 7*a* plots the simulated PDE**(*t*) activity for multiple photoisomerizations delivered to a single disc surface at time zero; the solid traces are ensemble mean responses for simulations in which at least 1000 photoisomerizations were delivered (e.g. 500 trials with two isomerizations per disc surface, and so on). The smallest red trace is for a single isomerization, and has been taken from figure 3*a*, with 4000 trials; the black traces are for two to nine isomerizations, and the remaining three red traces are for 10, 20 and 30 isomerizations. Each trial began with the specified integer number of photoisomerizations, distributed at random locations across the disc surface. One interesting aspect of the traces in figure 7*a* is a small degree of super-linearity for flashes delivering a few isomerizations per surface. For example, for two isomerizations the response peaks at 39.5 PDE** (28.0%), in comparison with 18.0 PDE** (12.8%) for a single isomerization. We chose not to run simulations for numbers of isomerizations other than those shown by the solid traces, because the computation time became long with large numbers of isomerizations, as a longer time was required for all the transducin molecules to be inactivated. Instead, we predicted the response waveforms for other intensities as described below.

With at least eight photoisomerizations per disc surface, the mean PDE**(*t*) response approaches its maximal level (representing the binding of two G*s to every PDE holomer) within about 100 ms of stimulus delivery. For intensities above this level, with the PDE fully bound, the peak level of free G* increases dramatically (not shown); for example, for 20 isomerizations per surface, the peak level of free transducin was 984 G* at 143 ms. Furthermore, when the PDE is fully bound in this way, our model assumes that the decline in G*(*t*) level results solely through decay from the PDE** state, which occurs at a rate *N*_E,surf_ *k*_E**_, where *N*_E,surf_ is the number of PDE holomers in the disc surface and *k*_E**_ is the rate constant of PDE** inactivation. Denoting the effective lifetime of R* as *T*_R*_, we see that each additional isomerization will produce *ν*_RG_ *T*_R*_ additional molecules of activated transducin, G*. As a result, at intensities that cause all PDEs in the disc surface to be doubly bound, each additional isomerization is predicted to elicit a rightward shift of the response falling phase of *ν*_RG_ *T*_R*_/(*N*_E,surf_ *k*_E**_). Substituting *ν*_RG_ = 1000 G* s^−1^, *T*_R*_ = 68 ms (as measured for our R* shut-off scheme), *N*_E,surf_ = 141 holomers per surface and *k*_E**_ = 5 s^−1^, we predict a rightward shift in the recovery phase of 96.4 ms for each additional photoisomerization per disc surface. For the simulations using 10, 20 and 30 isomerizations per surface, the mean rightward shift per extra photoisomerization was measured to be 94.1 ms. To estimate the expected responses for intermediate numbers of isomerizations (11–19 and 21–29), we interpolated between the simulations for 10 and 20 isomerizations, and between those for 20 and 30 isomerizations, by shifting and weighting appropriately. Those estimated responses are plotted as the blue traces in figure 7*a*, and will be used in the next section.

To put the predictions of figure 7*a* in perspective, we need to consider the level of PDE activity required to saturate the electrical response. From the analysis of the steady state, we find that 90% of the circulating current is suppressed for a hydrolytic activity of *β* = 66.6 s^−1^ (see §2.3), which is produced by about 2700 PDE** in the outer segment. Given that the PDE content of the outer segment is 2 × 10^5^ holomers, suppression of 90% of the circulating current would be achieved when the average level of PDE** in the outer segment is only approximately 1.3% of the total. On average, the fractional level in each disc surface will be the same (a mean of 1.8 PDE** per surface), but the actual number of PDE**s per disc surface will be Poisson distributed with this mean, and will therefore fluctuate widely.

### 3.7. Bright flash responses and the dominant time constant of recovery

From the results in the preceding section, we can calculate the predicted responses to bright flashes. To do this, we first need to know the number of disc surfaces per outer segment, which we calculate as around 1500 for a mammalian rod (approx. 750 discs, spaced at approx. 30 nm over a length of 22 µm). Because this number is quite large, it turns out to be sufficient to use the single-photon simulation results for flash intensities up to several hundred photoisomerizations per rod. For example, a just-saturating flash of 300 photoisomerizations per rod would deliver a mean of 0.2 photoisomerizations per disc surface. As a result, at that intensity only a very small proportion (less than 2%) of disc surfaces would receive multiple photoisomerizations, so that the response can be determined accurately from knowledge of the PDE** activity elicited by a single photoisomerization.

However, at higher intensities, we need a different approach. As set out in §2.6, we use the Poisson probability distribution, specifying the proportion of disc surfaces experiencing different numbers of photoisomerizations, in conjunction with the individual the traces in figure 7*a*, to determine the total PDE**(*t*) time-course throughout the outer segment, which we then use as the driving function for the downstream reaction cascade.

Figure 7*b* shows the calculated responses to a series of flashes at intensities that increase by 0.3 log_10_ units between traces (i.e. approximately doubling), from *Φ* = 1 to *Φ* = 16 000 photoisomerizations per rod. We repeated these calculations for many more flash intensities than are illustrated in figure 7*b*, and for each intensity, *Φ*, we measured the saturation time, *T*_sat_, defined as the time at which the falling phase of *R*(*t*) crosses the 90% level, corresponding to recovery of 10% of the dark current. We then plotted these values against flash intensity as the red curve in figure 7*c*, which gives *T*_sat_ as a continuous function of *Φ*. The dotted red line has a slope corresponding to 215 ms, and provides a good description of our simulated results up to about *Φ* = 3000 isomerizations per rod; for reasons that are not immediately obvious, the slope of this line is marginally higher than the time constant of PDE** shut-off, 1/*k*_E**_ = 200 ms, that we used in the simulations.

To compare this prediction of our model with experimental measurements, we have plotted the values presented by Burns & Pugh [32] (their fig. 4) for a WT mouse rod as the symbols in figure 7*c*. For intensities up to approximately 2000 isomerizations the red curve for our model provides a good description of their experiment, with each doubling of intensity eliciting a constant rightward shift (corresponding to the lower intensities in figure 7*b*). However, at higher intensities there is a significant discrepancy. Whereas the experimental measurements indicate a second time constant (dotted blue line) of 650 ms, our model in its present form predicts a steeper rise. As we shall return to in the Discussion, this defect in our model stems from the assumption (that we made for simplicity in the case of a single R* per disc surface) that shut-off of the free G* can be ignored.

## 4. Discussion

In the past, it has often been assumed that the rod's hetero-tetrameric PDE6*α*/PDE6*β* (with its two identical PDE6*γ* subunits) behaves as a pair of independent catalytic subunits. However, in the light of earlier reports [2,3] that the activation of the PDE is functionally asymmetric, together with the recent clear-cut demonstration that the singly bound form has negligible hydrolytic power [4], we felt it important to investigate the implications that this insight has for the rod's electrical response to light. We now examine the quantitative consequences that our model and simulations have for a more comprehensive understanding of phototransduction, especially in relation to the single-photon response and the continuous noise.

### 4.1. Amplification underlying the rod's single-photon response

Our model and simulations suggest that, during the rising phase of the rod's single-photon response, PDE**s are activated at a rate of *ν*_RE**_ ≈ 300 s^−1^, and that at the peak of the single-photon response there are approximately 18 PDE**s simultaneously active. In order to account for the experimentally observed amplitude of the single-photon response in mammalian rods, of approximately 4–5% of the dark current, we found it appropriate to set the PDE hydrolytic activity in the downstream reactions to be *β*_E**_ = 0.025 s^−1^, giving the amplitude of the ensemble mean of the simulated SPRs as 4.3% in figure 4*d*.

From the analysis of the phototransduction cascade [1], the magnitude of the parameter *β*_E**_ can be predicted from physical and biochemical parameters of the rod outer segment, using equation (2.4). That equation combines three parameters (*k*_cat_/*K*_m_, *V*_cyto_ and *B*_cG_) that can each be estimated from experimental measurements in the literature, though, it has to be said, with a fair bit of leeway in each estimate. For example, if we adopt values of *k*_cat_/*K*_m_ = 6 × 10^8^ s^−1^ M^−1^, *V*_cyto_ = 0.02 pL and *B*_cG_ = 2, then substitution into equation (2.4) predicts *β*_E**_ = 0.025 s^−1^, exactly as we used in the simulations. We do not consider any of the three underlying parameters to have been determined accurately for a mammalian rod *in vivo*, and we are simply showing that plausible values predict an appropriate magnitude for *β*_E**_. Thus, we conclude that an entirely reasonable set of physical and biochemical parameter values enables the downstream cascade of reactions to convert the simulated PDE** traces into electrical responses with properties closely emulating the experimentally measured single-photon responses in mammalian rods.

Viewed from a slightly different perspective, the amplification constant *A* of phototransduction is given by equation (2.3) as
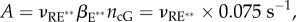
because *n*_cG_ = 3. Accordingly, our mean value of *ν*_RE**_ ≈ 310 PDE** s^−1^, for simulations with the standard set of parameters, predicts an amplification constant of *A* ≈ 23 s^−2^ for mouse rods, consistent with the upper end of estimates in the photoreceptor electrophysiology literature (§2.3). Therefore, we conclude that the PDE dimeric activation model, in conjunction with our standard set of parameters for lateral diffusion in the disc membrane and our suggested set of other physical and biochemical parameters, is able to account accurately for the amplification and rising phase kinetics of mammalian rod phototransduction.

### 4.2. Initial delay in the rising phase of the rod's flash response

In the ‘delayed Gaussian’ description of the rising phase of the rod's flash response given in equation (2.1), there is an in-built ‘effective delay time’, *t*_eff_, representing the cumulative effect of multiple short delays expected to arise from several steps in the cascade, as well as any filtering delay in the recording system [1]. Recently, Rotov *et al.* [7] examined the magnitude of the delay in rods and cones, and showed that the delay in frog rods at room temperature was around 10 ms longer than in cones. They noted that the experiments of Cobbs & Pugh [33], which used voltage clamp to circumvent the capacitive time constant, appeared consistent with this, in showing an irreducible delay of around 7 ms at room temperature, in salamander rods stimulated with very bright flashes. Rotov *et al.* [7] analysed a molecular model of PDE activation, based on the conventional concept of independent activation of PDE* subunits, and concluded that the longer delay in rods was paradoxical, and posed a problem for the existing model of diffusional interactions at the disc membrane.

As a result of our simulations, we suggest that the PDE dimeric activation model provides a compelling account for the existence of this additional delay stage in rods. Given an experimentally measured additional delay in rods of approximately 10 ms at room temperature, we would anticipate a delay of around 5–7 ms at mammalian body temperature. The results in figure 5*c*,*f* show that the magnitude of the time constant declines with increasing transducin activation rate and also with increasing density of PDE holomers. For our standard set of parameters, of *ν*_RG_ = 1000 G* s^−1^ and *C*_E_ ≈ 80 PDE holomers µm^−2^, the fitted time constant was *τ*_RE**_ ≈ 7 ms. If the PDE density were a little higher, at 125 PDE holomers µm^−2^, then the delay would drop to approximately 5 ms. The magnitude of this time constant will also depend on the assumed values of the lateral diffusion coefficients (primarily those of transducin and the PDE), and although we are not aware of any direct measurements of those parameters, the values that we have adopted are consistent with the literature (see table 1 of [13]).

Furthermore, we do not think there is a problem, as suggested in [7], regarding diffusional interaction at the disc membrane. Thus, the activation of G* by R* (in cones, as in rods) occurs at a rate far slower that the rate of diffusional contact, because of delays introduced by the microsteps of catalysis [27], such as the times taken to release GDP, to bind GTP and to release R*. Despite the lower surface packing density of PDE, the interaction of G* with PDE can occur faster (potentially at the diffusion limit) because this step simply involves binding rather than a series of catalytic microsteps. Nevertheless, in both cases some time may be required for rotational alignment.

### 4.3. Validity of parameter value: transducin activation rate

Of the parameter values that we have adopted, perhaps the one that is most likely to raise eyebrows is the rate of G* activation by a single R*, for which we have taken *ν*_RG_ = 1000 G* s^−1^, whereas several modelling studies in the literature have proposed approximately 300 G* s^−1^ at mammalian body temperature [9,23,24]. We now mention four lines of evidence supporting a value of this order of magnitude.

First, it conforms with measurements made from rods using light-scattering approaches [25–28]. With magnetically oriented frog rod outer segments, Vuong *et al.* [25] and Bruckert *et al.* [27] estimated the rate of transducin activation as around 1000 s^−1^ at room temperature, and with bovine rods, Kahlert & Hofmann [26] obtained 800 s^−1^ at 20°C. More recently, using purified bovine rod disc membranes reconstituted with purified transducin at different concentrations, Heck & Hofmann [28] reported a limiting rate of transducin activation per R* of 1300 G* s^−1^ at 34°C; adjustment to physiological conditions (37°C, and transducin density 3000 µm^−2^) converted this to *ν*_RG_ ≈ 1000 G* s^−1^.

Second, a value of this order of magnitude is needed to account for the amplitude of the rod single-photon response in our model of dimeric activation of PDE, when used in conjunction with values for the other parameters that are reasonably well-grounded in the literature. The most important of these other parameters are: the membrane density of PDE holomers, *C*_PDE_ = 80 µm^−2^; the lateral diffusion coefficient of transducin, *D*_G_ = 2.2 µm^2^ s^−1^; and the mean lifetime for an R*, of approximately 70 ms. In order to obtain more than a handful of PDE** molecules at the peak of the response, our simulations required *ν*_RG_ ≥ 500 G* s^−1^ (figure 5*c*), and our default value of *ν*_RG_ = 1000 G* s^−1^ generated 18 PDE** at the peak.

A third rationale comes from considering the variability of the single-photon responses. If only a few PDE** molecules were produced at the peak of the SPR, then the fluctuations in amplitude (and area) would be predicted to be very large, and so once again a rate of *ν*_RG_ < 500 G* s^−1^ would be inadequate. Our fourth line of evidence comes from considering the intensity at which the rod's bright flash response transitions from a first to a second dominant time constant. This will be presented in §4.9.

Clearly, there is a marked difference between the high rates of transducin activation, estimated in light-scattering experiments and required in our description, and the much lower rates estimated in biochemical experiments measuring nucleotide incorporation (e.g. [20]). For a proper understanding of vertebrate phototransduction, it will be important to resolve this discrepancy. For example, can it be demonstrated that the rate of transducin activation in disrupted rod outer segments is the same as the rate that applies in the intact cell *in vivo*? Another issue that deserves investigation is whether the catalytic activity of R* remains unchanged, from the early times (tens of millisecond) that are relevant to the electrical response and to the light-scattering measurements, out until the very late times (tens of seconds) at which biochemical measurements are made. In our recent analysis of the shut-off of R* activity during the single-photon response [9], we found it necessary to invoke a spontaneous 10-fold drop in R* activity prior to arrestin binding, in order to account for the late phase of the SPR obtained in rods of Arr^−/−^ mice. If a large drop in R* catalytic activity does occur for some reason, then this would have major ramifications for any measurements made at late times.

### 4.4. Validity of parameter value: membrane density of PDE

According to our simulated measures in figure 5*f*, the rate of PDE** activation has a fairly flat-topped peak for PDE membrane densities in the vicinity of 40–80 holomers µm^−2^. On the other hand, the initial delay is substantial at low densities, and declines with increasing holomer density. We would predict, therefore, that experiments with altered levels of PDE expression would reveal relatively subtle effects, with minor changes in amplitude and kinetics of the SPR; we are not aware of any experiments in the literature that test this prediction.

Values in the literature for the PDE holomer density, relative to rhodopsin, in bovine retina range from as high as 1 : 65 [34] down to 1 : 310 [35] (i.e. a PDE density of 80–400 holomers µm^2^). On the basis that a density of 80 holomers µm^2^ is sufficient to account for the onset phase of the response, and the expectation that increasing the PDE density would increase the noise resulting from spontaneous activation, we chose to standardize on the value reported by Pentia *et al.* [35]. Very similar estimates have been obtained for amphibian rods, with ratios to rhodopsin of 1 : 300 [36] and 1 : 270 [37].

### 4.5. Variability of the single-photon response

For the parameters that we adopted as standard, we determined the variability of the response to a single photon as the coefficient of variation, CV, both for the number of PDE** molecules active and also for the electrical response, and in both cases for the amplitude and also the area (integral) of the response. For PDE**, the coefficients of variation for the SPR were CV_area_ = 0.671 and CV_ampl_ = 0.545, while for the electrical response the corresponding values were CV_area_ = 0.561 and CV_ampl_ = 0.414.

These values are larger than reported in SPR experiments on mammalian rods, where CV_area_ is typically found to be no greater than 0.35. However, we suggest that underestimation of the experimental values could have arisen from the process of selection of singleton responses from the full set of responses comprising failures, singletons and responses to multiple hits. With the use of amplitude criteria to select singletons, we think it inevitable that the smallest singletons may get overlooked (as they are assumed to be failures) and that the largest singletons may likewise get overlooked (as they are assumed to be responses to multiple hits). Omission of the extrema will bias the sample and lead to underestimation of the true variance.

With this factor in mind, we regard the correspondence between simulation and experiment as adequate for preliminary modelling in which we have made no attempt to optimize parameters. We envisage that by adjusting various parameters in the model it would possible to achieve closer agreement with SPR experiments.

### 4.6. Unitary responses and the continuous noise

In order to test whether the stochastic occurrence of PDE** events could underlie the continuous noise recorded from mammalian rods, we subjected simulated stochastic PDE** events to the downstream cascade, and our results are shown in figure 6, for both WT and GCAPs^−/−^ genotypes. In panels *b*,*c*, we plotted the power spectral density predicted for the random activation of PDE** molecules, each having a stochastic lifetime determined by the GTPase reaction, and with the vertical scaling corresponding to a rate of one such spontaneous event per second per rod. In both genotypes, the fall-off at high frequencies has a slope corresponding to two filtering stages. The predicted zero-frequency asymptote and half-power frequency are 1.4 × 10^−6^ Hz^−1^ and 0.9 Hz for WT, and 5.2 × 10^−5^ Hz^−1^ and 0.45 Hz for GCAPs^−/−^.

Then we examined the spectral density measurements of dark noise from two studies in the literature (fig. 4*b* of Burns *et al.* [29] for mouse rods and fig. 14*b* of Baylor *et al.* [30] for monkey rods), and we determined the vertical scaling required for conformity between prediction and experiment. The required scaling corresponded to a mean rate of PDE** events of 40 s^−1^ per rod for GCAPs^−/−^ mouse (figure 6*b*), and to 11 s^−1^ per rod and 50 s^−1^ per rod for WT mouse and monkey rods, respectively (figure 6*c*). A shortcoming here is that the experimental recordings did not separate the continuous noise from the noise component arising from spontaneous photon-like events, but we think that this had relatively little impact because the photon-like event rate is very low in mammalian rods; for the future, it would valuable to make dedicated recordings isolating the continuous component of noise in mammalian rods. We think it possible that the WT mouse measurements may have underestimated the noise, because the noise in darkness was only marginally larger than that in saturating light, so that there may have been issues with the subtraction to obtain the biological noise. On balance, we will adopt 40 PDE** events s^−1^ per rod as a representative event rate for both genotypes.

A potential source of random activation of PDE**s would be the binding of spontaneously activated transducins (G*) to singly bound PDE* molecules (but see next section). For such a molecular mechanism, we can estimate the required rate (*ν*_G*_) of spontaneous transducin activation events required to account for the observed rate of PDE** events, if we know the fraction, *N*_E*_/*N*_E,_ of PDE molecules that are singly bound in the resting state. Thus, on the assumption (that is also made in our numerical simulations of lateral diffusion) that the fate of each G* depends simply on whether it contacts a singly bound PDE* or an unbound PDE, we can write4.1
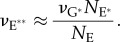


If we further assume that, in the dark resting state, the singly bound PDE*s had arisen solely as a result of spontaneously activated G* binding to unbound PDE, then the resting number of singly bound PDE*s is the product of their rate of formation and their mean lifetime4.2
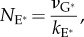
where *k*_E*_ is the rate constant of PDE* shut-off by GTPase activity. Combining equations (4.1) and (4.2), we obtain4.3
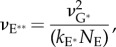
which predicts that the rate of PDE** events should increase as the square of the rate of spontaneous G* events.

When we substitute *N*_E_ = 2 × 10^5^ PDE holomers per rod, *k*_E*_ = 2.5 s^−1^ and *ν*_E**_ = 40 s^−1^ per rod, we obtain the required rate of spontaneous transducin activation as *ν*_G*_ = 4500 s^−1^ per rod. The ratio of estimated rates of spontaneous activation for transducin versus PDE** is therefore approximately 100-fold, which indicates that the amplitude of the electrical response elicited on average by a single transducin would be around 1% of that elicited by a single PDE**.

The above estimate of 4500 G* s^−1^ per rod represents an upper limit, because it is conceivable (*a*) that there might be other contributions to the resting PDE* level and/or (*b*) that there might be a source of spontaneous PDE** activation other than via G*. An example of the latter mechanism would be the spontaneous flickering release of inhibition by the γ-subunits; however, we would anticipate that such events would be quite fast, so that the noise they elicit would extend to frequencies higher than is observed in the spectrum of the rod's dark noise.

Even this upper limit, of 4500 G* s^−1^ over the entire outer segment, is not particularly high, and represents only 3 G* s^−1^ per disc surface. As the outer segment contains around 8 × 10^6^ transducins (at a density of 3000 µm^−2^), it corresponds to a G* activation rate of about 5 × 10^−4^ s^−1^ per molecule. For comparison, the rate of spontaneous release of GDP by bovine transducin has been measured in biochemical experiments as 1–2 × 10^−4^ s^−1^ at 37°C [38], so that the two estimates are within a factor of approximately 3 of each other. If we had instead assumed the lower event rate of 11 s^−1^ obtained for WT mouse rods to be correct, then our estimate for the G* activation rate would have halved, and would have been very close to the biochemical estimate.

If we multiply the rate of PDE** activation of 40 s^−1^ by the mean PDE** lifetime of 1/*k*_E**_ = 0.2 s, then we obtain an estimate for the mean number of PDE**s present in the outer segment under resting conditions, as 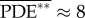. This number seems plausible in terms of the detectability of an SPR above the continuous noise, on the basis of the following simplistic calculation. Thus, the SD of the spontaneous fluctuations would be the square root of this, or approximately 3 PDE**s, giving peak-to-peak fluctuations of approximately 5 times greater, or 14 PDE**s. For comparison, from figure 4*c*, the SPR (at its peak) should generate a random number of PDE**s with a mean of 18 and an s.d. of approximately 10. As a result, for SPRs occurring at random times, it seems likely that the majority would be resolvable above the noise. Clearly, this is a simplistic calculation, and the detectability of the SPR could be analysed more rigorously in terms of the electrical response, once the spontaneous rate of unitary PDE** events has been established more accurately.

### 4.7. Comparison with other work on the molecular origin of rod continuous noise

For toad rods, it has been reported that the continuous noise in darkness arises from spontaneous fluctuations in PDE activity [39]. In an effort to rule out a role for spontaneous activation of transducin, that study applied extremely high concentrations of cyclic GMP (68 µM) in the dialysis solution bathing truncated toad rod outer segments, either in the presence or the absence of 10 µM GTP to allow or to block activation of transducin, and found no difference in the noise measured using a suction pipette. However, as far as we are aware, the potential role of spontaneous activation of transducin has not been investigated in mammalian rods, or at physiological concentrations of cyclic GMP in any rods. If experiments on mammalian rods were to confirm the absence of a role of transducin in generating the continuous dark noise, then our calculations in the preceding section, equations (4.1)–(4.3), would need to be rejected.

Our analysis was undertaken in light of the preliminary report of Qureshi *et al.* [3] and as an extension of their detailed analysis [4], and it therefore has many features in common with that work. Their paper concentrates on the biochemical evidence, on simulations of PDE activation at the disc membrane, on noise immunity and on potential molecular mechanisms underlying the functional asymmetry. By contrast, our paper concentrates on the electrical response of the intact rod photoreceptor. But both studies concur on the finding that dimeric activation of PDE is expected to provide substantial immunity against the effects of spontaneous activation of transducin, even when such activation occurs at quite a high rate.

One difference in the numerical simulations is that we have used a simpler (and computationally faster) description of lateral diffusion and intermolecular contacts than the more comprehensive approach adopted by Qureshi *et al.* [4], and as a result, we have been able to average over a much larger number of stochastic simulations. Another difference is that they introduced finite on- and off-rates (*k*_on_ and *k*_off_) for the binding of G* to PDE and to PDE*; in particular, they reduced the value of *k*_on_ for the second reaction, in light of their measurement of a higher dissociation constant in that case. Instead, we chose not to implement a finite value of *k*_on_ for two reasons. First, we feel that specifying such rates on the basis of equilibrium measurements introduces uncertainty. Second, in preliminary trials, where we did simulate a lowered *k*_on_ for binding to form PDE**, we found that the main effect appeared to be an increase in the delay prior to the ramp-like rise of PDE** (data not shown), and that this increase was beyond what is expected from experimental recordings. The effect deserves investigation in future work, once kinetic measurements of *k*_on_ and *k*_off_ are available, but it is beyond the scope of the present paper.

### 4.8. Dark PDE activity (*β*_Dark_)

From the mean number of PDE**s active in the dark resting state, in conjunction with the hydrolytic activity per PDE**, we can readily estimate the mean dark level of cGMP hydrolysis generated by the stochastic activation mechanism. By setting 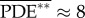, and using our standard value of *β*_E**_ = 0.025 s^−1^, we obtain a hydrolytic rate of *β*_spont_ ≈ 0.2 s^−1^, far smaller than the resting dark rate of hydrolysis, of *β*_Dark_ = 4 s^−1^. As a result, we can conclude that *β*_Dark_ is not set by stochastic activation of the PDE to its fully activated PDE** state.

The two most likely alternatives are that *β*_Dark_ results from the residual activity of either the unbound PDE holomers or the singly bound PDE* molecules, for example, by flickering relief of inhibition by the γ-subunit(s). Up until now, we have ignored any hydrolytic activity of the singly-bound PDE*, and we regard this simplification as entirely justifiable in calculating the kinetics of activation, because Qureshi *et al.* [4] reported its activity to be no more than 2.5% that of PDE**. Nevertheless, it might contribute to *β*_Dark_. If PDE* were the primary source of the resting hydrolytic rate, then its residual activity would need to be *β*_Dark_/*N*_E*_ ≈ 4 s^−1^/1800 = 0.0022 s^−1^. This would be approximately 9% of the hydrolytic rate of the fully active PDE** (*β*_E**_ = 0.025 s^−1^), a level considerably higher than the reported upper limit of 2.5% [4]. If, instead, unbound PDE holomers were the primary source of resting hydrolytic activity, then their activity per holomer would need to be *β*_Dark_/*N*_E_ ≈ 4 s^−1^/2 × 10^5^ = 2 × 10^−5^ s^−1^, or around 1/1000 that of the fully activated PDE**. We have no basis for rejecting residual activity of 0.1%, and so it is plausible that *β*_Dark_ represents the very low residual activity of the unbound PDE holomers, or a combination of residual activity of both PDE and PDE*.

### 4.9. Saturating flashes

Using our model, we predicted the relationship expected between flash intensity *Φ* and the time *T*_sat_ that the response to that flash remains in saturation (figure 7*c*, red trace). For intensities up to *Φ* ≈ 3000 photoisomerizations per rod, our model predicted exactly the relationship reported in the literature [29,40,41], with the slope in semi-logarithmic coordinates representing a ‘dominant time constant’ of approximately 200 ms. However, at very high intensities there is a discrepancy between the predictions of our model, as it stands, and experimental results in the literature. Thus, the experiments typically show a second straight-line region in semi-logarithmic coordinates, whereas our model predicts a relationship that is straight-line in linear coordinates, with upward curvature in the semi-logarithmic axes of figure 7*c*. We interpret this discrepancy to indicate a fault in our assumption that activated transducin (G*) can only decay while it is bound to PDE, because it is this assumption that leads to the linear dependence of *T*_sat_ on *Φ* at very high intensities. We made that assumption as a simplification to avoid introducing an additional parameter, and the simplification has a negligible effect when there is only a single R* per disc surface, because the level of free G* is normally quite small. However, at higher intensities that saturate the binding of G* to PDEs, the level of free G* rises, and hence the lifetime of free G* needs to be taken into account.

The second, longer ‘dominant time constant’ (of 600–800 ms) observed in electrophysiological measurements on mouse rods has been proposed to reflect the time constant of such decay of free G* *in vivo*, on the basis of experiments incorporating RGS9-2 into rods [42]. We concur with that interpretation, and note that it conforms with the biochemical finding that, in the presence of the RGS domain of RGS9 but in the absence of PDE*γ*, the rate of GTP hydrolysis by activated transducin is around 1 s^−1^ [43] (in contrast to the much slower rate for purified transducin α-subunits, of around 0.05 s^−1^ [44]). Accordingly, the two ‘dominant time constants' measured electrophysiologically are likely to reflect the time constants of decay of doubly bound PDE** and free G*, respectively. We will examine the detailed consequences of introducing a finite rate of inactivation for free G* in future simulations.

Importantly, this interpretation provides the fourth line of support (see §4.3) for our use of a G* activation rate of at least 1000 G* s^−1^ per R*. The transition intensity, *Φ*_trans_, for the change from smaller to larger dominant time constant should correspond to the creation of just enough G*s to bind both sites on the PDE holomers. Hence, the rate of G* activation required to account for the transition intensity should satisfy4.4
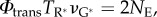
where *Φ*_trans_ has been measured as approximately 4000 R* per rod [32], the R* lifetime is *T*_R*_ ≈ 0.07 s, and the rod contains *N*_E_ = 2 × 10^5^ PDE holomers. Consequently, the required rate of G* activation is calculated as *ν*_G*_ ≈ 1400 G* s^−1^ per R*, even higher than the value we have adopted.

### 4.10. Cone phototransduction

Our analysis so far has been directed solely towards rods, but we can suggest some consequences for cones, based on the fact that the cone PDE6 is composed of a pair of identical *α*’ subunits (encoded by *PDE6C*) and is presumably a symmetrical molecule. It is therefore plausible to think that activation of the cone PDE by transducin may not display functional asymmetry, and that the two catalytic subunits may instead operate independently of each other, as has in the past been envisaged to be the case for the rod PDE. However, as we do not know the precise molecular mechanisms involved in activation, this suggestion for cones remains speculative. Nevertheless, if it were the case, then cones could be viewed as having avoided the combination of advantages and disadvantages that are afforded to rods by dimeric activation.

Such a scenario could well explain at least two known differences between cones and rods, and it might predict other differences as well. First, it would explain the shorter delay in the rising phase of the response that has recently been characterized [7]. Second, it would readily account for the much higher level of continuous noise observed in cones (as, for example, reported in turtle cones [19] and monkey cones [45]). Third, it might contribute to the higher dark rate of PDE activity in cones, and therefore account in part for their smaller and faster responses to dim flashes. If all other factors were equal, independent activation of subunits would predict (perhaps counterintuitively) a higher efficacy of coupling from transducin to PDE in cones than in rods, with potentially every G* being effective, rather than with only approximately 60% being effective in the case of dimeric activation (see trace for efficacy in figure 5*c*). In theory, this factor could scale the amplification constant of cones relative to rods by up to 1/0.6, thus giving an increase of greater than 50%.

### 4.11. Consequences

If our interpretations in this study are broadly correct, then they have multiple implications for the reassessment of previous analysis and modelling of vertebrate phototransduction, and they also enable us to make suggestions for future work on refining and extending the concepts.

The first matter to consider for reassessment is the report in the literature that the rate of transducin activation in amphibian rods at room temperature is as low as 120–150 G* s^−1^ [20]. As mentioned in §4.3, this claim was based on the untested assumptions that the rate of transducin activation by R* remains undiminished out until the tens of seconds required to make the measurements of GTP*γ*S binding, and that the activation rate is the same in disrupted rod outer segments as it is *in vivo*. If, however, the rate of transducin activation at late times in disrupted outer segments differs from that at early times *in vivo*, then the biochemical measurements could potentially be very misleading.

Likewise, we suggest that all previous modelling of vertebrate phototransduction based on this value (or on its extrapolation to mammalian body temperature) should be reassessed in light of the critical question of whether the parameters of the model were well constrained. As discussed recently by Gross *et al.* [46], ‘Ill-constrained models, even if they accurately describe aspects of the data, can lead to ambiguous and even false inferences.' We certainly make no claim that the preliminary parameters that we have chosen for our present description are well constrained, or that our model is complete. But we think it unlikely that previous models can be considered to have been well constrained either if they used an erroneously low value for the rate of transducin activation or if they overlooked an important step in the molecular process of activation of the PDE.

For the future, we intend to investigate whether it is possible to extend the predictions of the dimeric PDE activation model to account more comprehensively for experimental measurements in the literature. For this present analysis, we concentrated on tests with an assumed rate of G* activation per R* of 1000 G* s^−1^, but it is possible that the true value *in vivo* might be even higher. If so, this would increase the mean number of PDE**s produced during the response to a single photon, and thereby lower the variability (i.e. lower the CV_area_). At the other extreme of the intensity range, for the predicted responses to very bright flashes, we anticipate that by incorporating inactivation of free G* it may well be possible to account quantitatively for the second, longer ‘dominant time constant’. In addition, as set out in §4.9, we think it likely that a higher rate of G* activation may make it easier to account for the relatively low transition intensity for the change from the first to the second dominant time constant.

Overall we conclude that, by breaking free of the unsubstantiated assumption that rod PDE subunits activate independently, it has been possible to obtain a more realistic model of phototransduction in rods, and that further refinement will be possible in the future.

## Supplementary Material

Appendix

## Data accessibility

The WalkMat package of Matlab computer code used to run the stochastic simulations and to integrate the downstream reactions in support of the findings in this article has been deposited in Dryad at (http://dx.doi.org/10.5061/dryad.n774460) [10].
