# Supplementary material for: Implications of dimeric activation of PDE6 for rod phototransduction
Source: Open Biol. 2018 Aug 1;8(8):180076. doi: 10.1098/rsob.180076 (PMC6119862; doi:10.1098/rsob.180076)
Supplement: Appendix [file rsob180076supp1.pdf]

Supplementary Material for

DOI: 10.1098/rsob.180076

Implications of dimeric activation of PDE6 for rod phototransduction

Trevor D. Lamb[1], Martin Heck[2] and Timothy W. Kraft[3]

[1]Eccles Institute of Neuroscience, John Curtin School of Medical Research,
The Australian National University, ACT 2600, Australia,
[2]Institut für Medizinische Physik und Biophysik, Charité - Universitätsmedizin Berlin,
corporate member of Freie Universität Berlin, Humboldt-Universität zu Berlin,
and Berlin Institute of Health, Berlin, Germany, and
[3]Department of Optometry and Vision Science, University of Alabama at Birmingham,
Birmingham, AL, USA.

Equations for the downstream phototransduction cascade

We employed a well-established model of phototransduction, with Ca^{2+}-mediated feedback onto cyclic GMP concentration via GCAPs and guanylyl cyclase, fundamentally the same as used in numerous previous investigations, and as set out explicitly in [9]. We included longitudinal diffusion of cGMP and Ca^{2+} within the outer segment [23], so that the expressions for cGMP and Ca^{2+} each required a partial differential equation (p.d.e.). Those two differential equations and the remaining equations for the downstream reactions are as follows. Most variables include the spatial coordinate (x) as well as time (t). The parameters in these equations are defined in Table 2, where their values are also listed.

cGMP p.d.e.:
$$\frac{\partial cG(x,t)}{\partial t} = \varphi_{cG}(x,t) + D_{cG}\frac{\partial^2 cG(x,t)}{\partial x^2} \qquad (A.1)$$

Calcium p.d.e.:
$$\frac{\partial Ca(x,t)}{\partial t} = \varphi_{Ca}(x,t) + D_{Ca}\frac{\partial^2 Ca(x,t)}{\partial x^2} \qquad (A.2)$$

cGMP net formation:
$$\varphi_{cG}(x,t) = \alpha(x,t) - \beta\, cG(x,t) \qquad (A.3)$$

Calcium net influx:
$$\varphi_{Ca}(x,t) = \frac{\frac{1}{2} f_{\mathrm{Ca}}\, L\, j_{\mathrm{cG}}(x,t) - L\, j_{\mathrm{ex}}(x,t)}{\mathcal{F}\, B_{\mathrm{Ca}}\, V_{\mathrm{cyto}}} \qquad (A.4)$$

Cyclase:
$$\alpha(x,t) = \frac{\alpha_{\mathrm{max}}}{1+\{Ca(x,t)/K_{\mathrm{GCAP}}\}^{m_{\mathrm{GCAP}}}} \qquad (A.5)$$

Channels:
$$L\, j_{\mathrm{cG}}(x,t) = J_{\mathrm{cG,\,max}}\frac{cG(x,t)^3}{cG(x,t)^3 + K_{\mathrm{cG}}{}^3} \qquad (A.6)$$

Exchanger: $$L\, j_{\mathrm{ex}}(x,t) \;=\; J_{\mathrm{ex,\,max}} \frac{Ca(x,t)}{Ca(x,t) + K_{\mathrm{ex}}} \qquad \text{(A.7)}$$

OS current: $$J(t) \;=\; \int_L \{\, j_{\mathrm{cG}}(x,t) + j_{\mathrm{ex}}(x,t) \,\}\, \mathrm{d}x\,. \qquad \text{(A.8)}$$

The membrane currents above are expressed as current *density* per unit length of outer segment, $j_{cG}(x,t)$ and $j_{ex}(x,t)$, but for convenience have been written in the form $L\, j(x,t)$, with units of current over the entire outer segment length, L.

Hydrolytic activity at location of photoisomerization

The PDE hydrolytic activity β in Eqn (A.3) above was set to

PDE activity (resting): $$\beta \;=\; \beta_{\mathrm{Dark}} \qquad \text{(A.9)}$$

at all longitudinal locations except where a photoisomerisation occurred.

For numerical simulation, we used spatial elements of finite width, δx. In an element that received a photoisomerisation (typically at $x = 0$), we set

PDE activity (isomerisation): $$\beta \;=\; \beta_{\mathrm{Dark}} + \beta_{\mathrm{E^{**}}} \frac{L}{\delta x} PDE^{**}(t) \qquad \text{(A.10)}$$

meaning that the photon-induced PDE activity was distributed over the width δx of the element. The number of activated molecules, $PDE^{**}(t)$, was obtained from the stochastic simulations described in the paper.

Boundary conditions at ends

The boundary conditions at each end of the outer segment are of the reflective (zero flux) kind. They apply for both cGMP and Ca^{2+}, and can be written as:

At each end of OS: $$\frac{\partial cG(x,t)}{\partial x} \;=\; 0 \;=\; \frac{\partial Ca(x,t)}{\partial x}\,. \qquad \text{(A.11)}$$

Bright flash responses

For bright flashes, we used a single compartment (i.e. a longitudinal element $\delta x = L$) and we dispensed with Eqns (A.1), (A.2), (A.9) and (A.11).

Computer code

The Matlab computer code used for solving these equations is part of the ‘WalkMat’ package, and has been deposited in Dryad with the link given under Data Accessibility.
